# Genetic comparisons of fall armyworm populations from 11 countries spanning sub-Saharan Africa provide insights into strain composition and migratory behaviors

**DOI:** 10.1038/s41598-019-44744-9

**Published:** 2019-06-05

**Authors:** Rodney N. Nagoshi, Georg Goergen, Hannalene Du Plessis, Johnnie van den Berg, Robert Meagher

**Affiliations:** 1 Center for Medical, Agricultural and Veterinary Entomology, United States Department of Agriculture-Agricultural Research Service, Gainesville, Florida United States of America; 2 International Institute of Tropical Agriculture (IITA), Cotonou, Benin; 3 Unit for Environmental Sciences and Management, North-West University, Potchefstroom, South Africa

**Keywords:** Animal migration, Agricultural genetics, Haplotypes

## Abstract

The recent discovery of fall armyworm (*Spodoptera frugiperda*, J.E. Smith) in Africa presents a significant threat to that continent’s food security. The species exhibits several traits in the Western Hemisphere that if transferred to Africa would significantly complicate control efforts. These include a broad host range, long-distance migratory behavior, and resistance to multiple pesticides that varies by regional population. Therefore, determining which fall armyworm subpopulations are present in Africa could have important implications for risk assessments and mitigation efforts. The current study is an extension of earlier surveys that together combine the collections from 11 nations to produce the first genetic description of fall armyworm populations spanning the sub-Saharan region. Comparisons of haplotype frequencies indicate significant differences between geographically distant populations. The haplotype profile from all locations continue to identify Florida and the Caribbean regions as the most likely Western Hemisphere origins of the African infestations. The current data confirm the uncertainty of fall armyworm strain identification in Africa by genetic methods, with the possibility discussed that the African infestation may represent a novel interstrain hybrid population of potentially uncertain behavioral characteristics.

## Introduction

Since its detection in São Tomé and Príncipe in April 2016^1^, fall armyworm (*Spodoptera frugiperda* [J.E. Smith]) has been found throughout sub-Saharan Africa^2–4^. Economic damage has been primarily in corn (maize) with costs estimated to be in the billions USD^5^. The potential for losses in other crops has yet to be determined but is expected to be significant given fall armyworm behavior in the Western Hemisphere where it is capable of feeding on over 80 plant species, periodically causing significant economic damage to rice, sorghum, millet, soybean, wheat, alfalfa, cotton, turf, and feed grass crops^6^. This broad host range is in part due to the existence of two subpopulations that differ in their distribution on host plants^7,8^. Other characteristics that make fall armyworm particularly problematic for agriculture is it’s migratory capability^9,10^ and the propensity of the species to develop resistance to pesticides, including those from *Bacillus thuringiensis* that are frequently used in organic farming or incorporated into genetically modified crops^11–15^. Characterizing the subpopulations and traits present in the African infestation is in its early stages.

Genetic markers derived from portions of the mitochondrial *Cytochrome Oxidase Subunit I* (*COI*) and the sex-linked *Triosephosphate isomerase* (*Tpi*) genes have been instrumental in studying the complexities of fall armyworm population dynamics^16,17^. The species can be divided into subpopulations historically identified as “host strains”^7^, with the “rice-strain” predominating on millet and pasture grass species while the “corn-strain” preferring corn and sorghum^18–20^. These observations indicate that the strain composition of the Africa fall armyworm infestation could have important ramifications for assessing crops at risk. However, such analysis is complicated by the morphological similarity of the two strains, which leaves mitochondrial or Z-chromosome-linked (such as *Tpi*) markers the only available means of strain identification^17,21^. The correspondence between host plant and markers has been demonstrated for populations throughout the Western Hemisphere but is not absolute, suggesting some inaccuracy in the markers or plasticity in host plant choice^19,21,22^. Nevertheless, the association of the *COI* and *Tpi* markers with host strains has been sufficiently consistent to demonstrate marker-defined differences in female pheromone constitution, mating behavior, and mating compatibility^23–26^. Several studies have shown that the strains are capable of productive hybridization in the laboratory and field, though this appears to occur at reduced frequency consistent with evidence of pre-mating barriers^8,25–28^.

A comparison of mitochondrial haplotype frequencies divides the Western Hemisphere corn-strain populations into two geographically distinct groups designated the FL- and TX-types^29,30^. The TX-type profile is found throughout South America, Mexico, and most of the United States, with the FL-type limited to Florida, the Caribbean, and the eastern coast of the United States^9,31,32^. These markers allowed a mapping of fall armyworm migration patterns in North America^33^. Fall armyworm does not survive prolonged freezing temperatures so permanent populations in North America are limited to southern Florida and southern Texas, with more northern infestations due to migration. Over the course of the agricultural season large populations of fall armyworm move progressively northward facilitated by favorable southerly winds and the parallel progression of corn agriculture, covering a distance of several thousand kilometers in 1–3 months^10,34^. This migratory capability could explain the apparent rapid spread of fall armyworm in Africa if similar conditions are present and would present a considerable challenge for developing pest management programs.

This collection of genetic markers was applied to fall armyworm collections from the western Africa country of Togo^35^. Only a small number of *COI* and *Tpi* haplotypes were found, with haplotype frequencies most indicative of the FL-type, suggesting a Western Hemisphere origin from Florida or the Caribbean. A subsequent study expanded this survey to fall armyworm in central (Democratic Republic of Congo, Burundi) and eastern (Kenya, Tanzania) Africa as well as the islands of São Tomé and Príncipe, which lie approximately 250 km off the western Africa coast^36^. The same set of *COI* and *Tpi* haplotypes present in Togo were found in all locations, consistent with a single introduction and subsequent dispersal to or from these sites through natural migration or by contamination of trade goods. However, some significant differences were found in haplotype frequencies between regions indicating that the magnitude of the fall armyworm movements may not be sufficient to maintain a homogeneous population. If true, this would suggest that the movement of fall armyworm throughout the northern sub-Saharan region involves the dispersal of relatively small numbers rather than the large migratory populations annually occurring in North America.

One example of regional haplotype differences was exhibited by the *COI* polymorphisms diagnostic of fall armyworm strains. This was surprising since all specimens came from corn-strain preferred hosts and so were expected to be predominated by the corn-strain haplotypes. The *COI* markers for both strains were present in the surveyed locations, a finding consistent with recent reports from Uganda and South Africa^37,38^. The frequency of the corn-strain *COI* variant was significantly higher in Togo and São Tomé and Príncipe than in the more eastern sites, where the rice-strain *COI* haplotype uncharacteristically predominated. However, when the same collections were analyzed for the strain-defining *Tpi* exon segment, the corn-strain *Tpi* haplotypes were present at >90% frequency at all locations. The minority *Tpi* haplotype carried the rice-strain marker but had additional sequence variations not yet found in Western Hemisphere populations^36^. This discrepancy between the *COI* and *Tpi* strain identification combined with the uniqueness of the rice-strain *Tpi* haplotype makes the strain composition of the African populations uncertain and brings into question the accuracy of the *COI* strain markers in Africa. This is of concern as recent studies based solely on *COI* have concluded the presence of the rice-strain in multiple African locations^2,37,38^, which could influence assessments of crops at risk. It is therefore relevant to further assess the accuracy of the *COI* marker for strain identification in African populations.

The objectives of this work were to expand the genetic survey to now include southern Africa, an additional year of collections in Togo, and new sites in central Africa that when combined with earlier studies provide a near continent-wide genetic comparison of African fall armyworm populations. This description of fall armyworm distribution patterns during the first two years of its detection in Africa can be used to identify future changes as the populations further equilibrate. In addition, sequence comparisons of a highly variable *Tpi* intron segment were performed to better assess the level of genetic variation in the different fall armyworm populations and to confirm the strain-identity of the unique *Tpi* haplotype found in Africa (currently designate as a rice-strain marker). The implications of these results on the likely number of introductions into the African continent and the strain composition of the African populations are discussed.

## Results

### Host strains in Africa

We previously analyzed fall armyworm populations from six African nations in the northern sub-Saharan region for genetic variation in the mitochondrial *COI* and sex-linked Tpi genes^36^. We now extend this work to include five additional countries that expand the surveyed region westward to Ghana and southward to South Africa as well as providing a second year of collections from Togo and additional specimens from the Democratic Republic of Congo (Table 1, Supplementary Fig. S1).

**Table 1:** Source information for fall armyworm specimens.

Label	Country	Regions (n)	Year	Collector/Reference
BUR	Burundi	Multiple sites (38)	2016–17	Nagoshi *et al*.^36^
CAR	Cen Afr Rep^a^	Ombella-M’poko (33)	2017	S. Ngarassem
CHA	Chad	Multiple sites (19)	2017	N. A. Doyam
GHA	Ghana	Multiple sites (44)	2016	G. Goergen
nDRC	DR Congo^b^	Sud-Ubangi (27)	2017	Nagoshi *et al*.^36^
sDRC	DR Congo^b^	Haut-Katanga (72)	2017	Nagoshi *et al*.^36^
KEN	Kenya	Multiple sites (55)	2017	Nagoshi *et al*.^36^
SAf	South Africa	Multiple sites (74)	2017	H. Du Plessis
STP	Sao Tome^c^	Multiple sites (22)	2016	Nagoshi *et al*.^36^
TAN	Tanzania	Morogoro (69)	2017	Nagoshi *et al*.^36^
TOGa	Togo	Multiple sites (89)	2016	Nagoshi *et al*.^36^
TOGb	Togo	Lomé (340)	2017	Meagher *et al*.^51^
ZAM	Zambia	Serenje (74)	2017	M. Rice
ARG	Argentina	Multiple sites (153)	2011–12	Murua *et al*.^43^ Nagoshi *et al*.^9^
BRA	Brazil	Multiple sites (76)	2008	Nagoshi *et al*.^30^
PR	Puerto Rico	Multiple sites (236)	2009–12	Nagoshi *et al*.^31,32^
TX	Texas, USA	Multiple sites (414)	2008–15	Nagoshi *et al*.^9,31^
FL	Florida, USA	Multiple sites (783)	2008–15	Nagoshi et al.^9,31^

^a^Central African Republic. ^b^Democratic Republic of the Congo. ^c^São Tomé and Príncipe.

**Figure 1:**
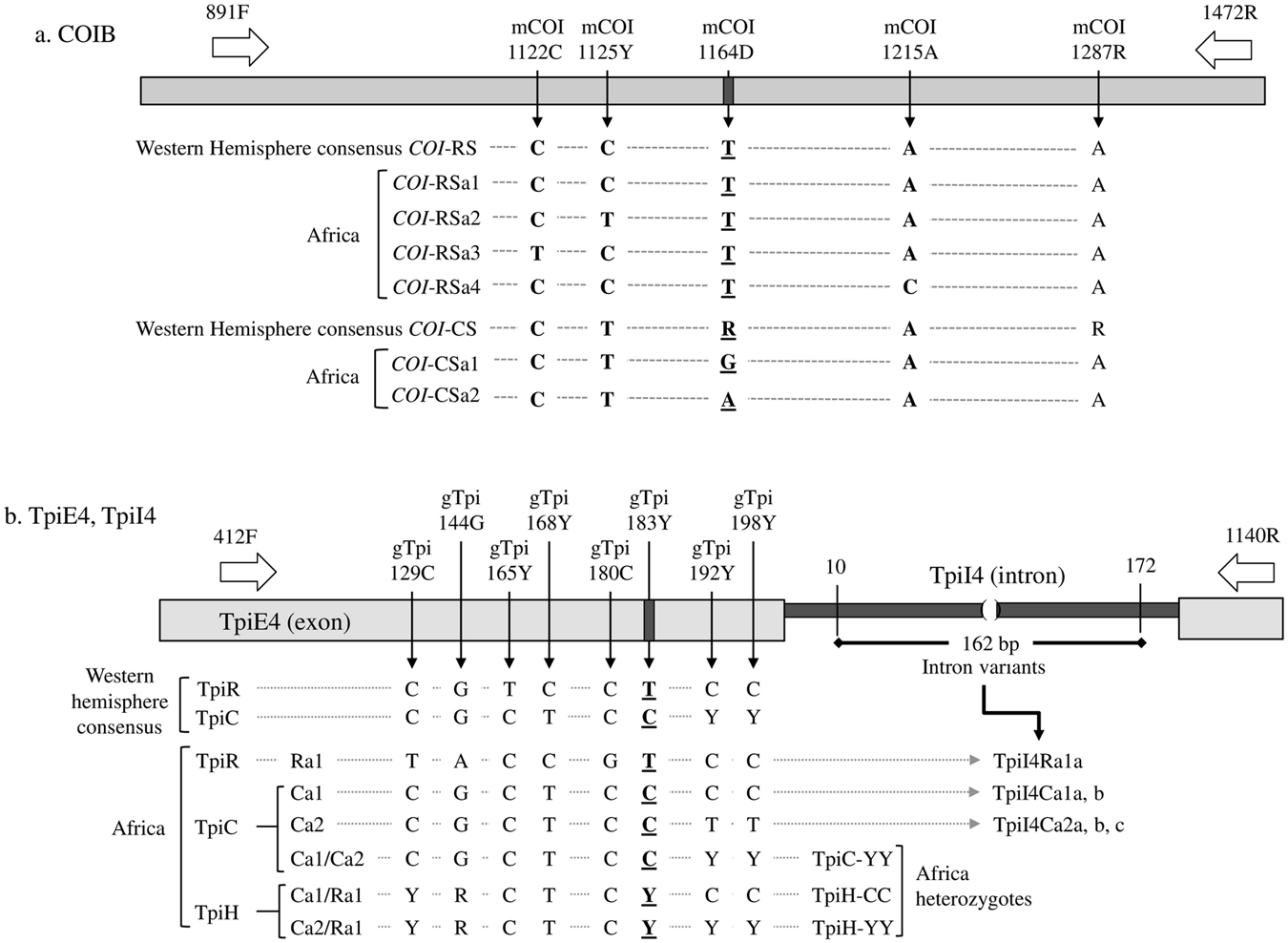
Diagrams of the COIB, TpiE4, and TpiI4 gene segments with respect to consensus Western Hemisphere sequences and the haplotypes observed in Africa. (**a**) The mCOI1164D site defines COI-based strain identity. Other polymorphisms define haplotypes. (**b**) The gTpi183Y site defines Tpi-based strain identity. Seven polymorphic exon sites differentiate the consensus strain-specific Western Hemisphere sequences and the three TpiE4 haplotypes from Africa that can be further subdivided by sequence variations in the 162-bp TpiI4 intron segment.

The *COI* and *Tpi* genes contain single nucleotide polymorphisms (SNPs) that are diagnostic of fall armyworm host strain identity in Western Hemisphere populations (Fig. 1). Specifically, site mCOI1164D (see Methods for nomenclature) is diagnostic of strain identity with T_1164_ identifying the rice-strain *COI*-RS haplotype group and either A_1164_ or G_1164_ indicative of the corn-strain *COI*-CS label (C_1164_ has not been found in this species). Polymorphisms at five other sites (mCOI1125Y, mCOI1176Y, mCOI1182Y, mCOI1197R, mCOI1216W) also show a strong strain bias in Western Hemisphere populations (Supplementary Fig. S2). Strain-diagnostic markers are also found in a portion of the fourth exon (TpiE4) of the coding region in the nuclear and sex-linked *Tpi* gene^17^. TpiE4 contains three sites (gTpi165Y, gTpi168Y, and gTpi183Y) that are strain-specific in Western Hemisphere populations, with gTpi183Y used as the diagnostic marker to define the corn-strain (TpiC) or rice-strain (TpiR) identity (Fig. 1b). Because *Tpi* is on the *Z*-chromosome, heterozygosity (TpiC/TpiR) is possible in male specimens and is denoted as TpiH.

**Figure 2:**
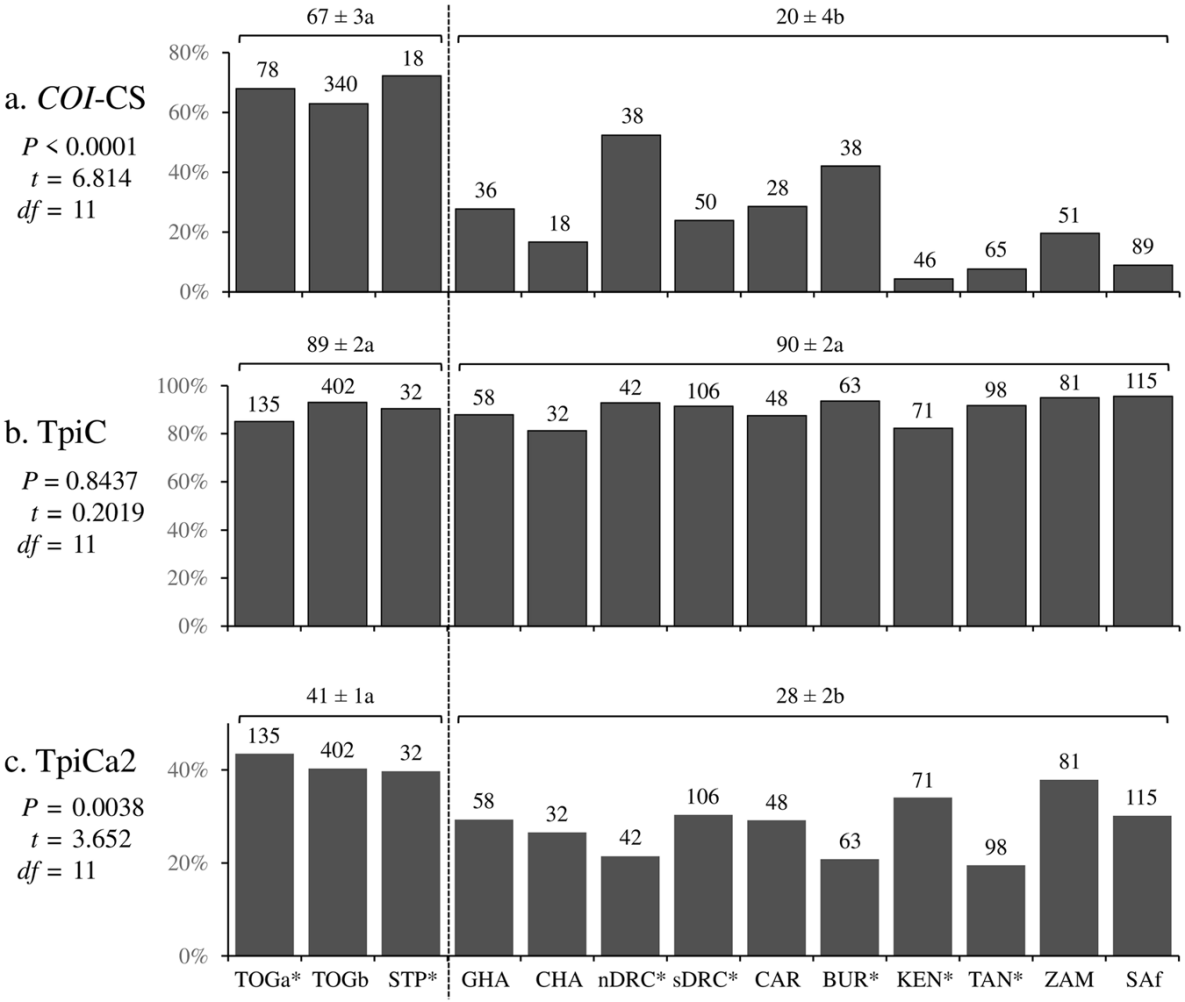
Comparisons of *COI* and *Tpi* haplotype frequency in 13 fall armyworm collection sets, including seven from a previous study^36^ (*). The Togo and São Tomé and Príncipe collections were compared with that from the rest of Africa by two-tailed *t*-test analysis. The mean percent ± standard deviation is indicated over the horizontal bars, with different lower-case letters indicating statistical significance. (**a**) Frequency of the *COI*-CS strain diagnostic marker based on analysis of the COIB segment. Number of specimens indicated above columns. (**b**) Frequency of the TpiC strain diagnostic marker from the TpiE4 exon segment adjusted for the contribution of heterozygotes. Estimated number of chromosomes tested indicated above columns. (**c**) Frequency of the TpiCa2 variant from the TpiE4 exon segment adjusted for the contribution of heterozygotes. Estimated number of chromosomes tested indicated above columns.

**Figure 3:**
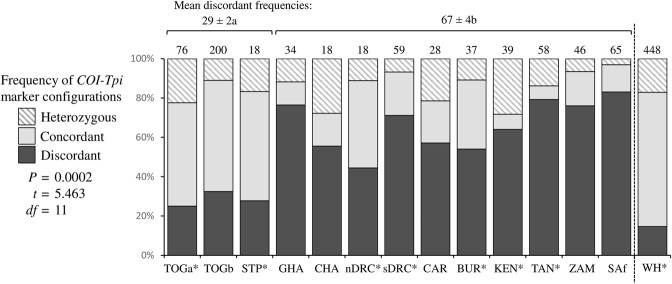
Frequencies of *COI* and *Tpi* strain marker configurations in Africa compared to pooled data from the Western Hemisphere. Configurations are categorized as Concordant (*COI*-CS TpiC, *COI*-RS TpiR), Discordant (*COI*-CS TpiR, *COI*-RS TpiC), and Heterozygous (*COI*-CS TpiH, *COI*-RS TpiH). Number of specimens is indicated above columns. Mean frequencies of the Discordant configurations from Togo and São Tomé and Príncipe collections were compared with that from the rest of Africa by two-tailed *t*-test analysis.

The majority of the collections from Togo and São Tomé and Príncipe express the *COI*-CS marker with a mean frequency that is significantly different from that of the rest of the continent where the *COI*-RS rice-strain marker predominates (Fig. 2a). In contrast, the *Tpi* rice-strain indicator, TpiR, was in the minority in all locations, representing 1% of observed haplotypes. Taking TpiH heterozygotes into account (described in Methods) resulted in an overall mean of about 10% TpiR frequency on a per chromosome basis with the corn-strain TpiC marker predominating in all locations with no apparent significant differences between locations (Fig. 2b). These observations indicate that the *COI* and *Tpi* strain markers must be in disagreement in much of Africa. This can be seen by comparing the frequency of specimens expressing a discordant (*COI*-CS TpiR or *COI*-RS TpiC) configurations (Fig. 3). The discordant configurations are considerable or predominate at all locations with the exceptions of Togo and São Tomé and Príncipe, where the concordant configurations are the majority at levels typical of the Western Hemisphere. As expected from the rarity of the TpiR marker in Africa nearly all of the discordant specimens (99%, 367/371) are *COI*-RS TpiC.

### Characterization of COIB haplotypes

A total of 616 sequences from this study were combined with earlier data^36^ for a total of 905 African sequences from which six COIB haplotypes were identified (Table 2). All polymorphisms are single-base changes that do not alter the presumptive amino acid sequence. The Africa populations were predominated by two COIB haplotypes. The *COI*-CSa1 form made up 99% (372/376) of the *COI*-defined corn-strain group (*COI*-CS) while *COI*-RSa1 was 95% (502/529) of the rice-strain (*COI*-RS) subpopulation (Table 2). Both are identical to the predominant COIB haplotypes in the Western Hemisphere (Supplementary Fig. S2). Four additional rare haplotypes were found that include the *COI*-CS variant *COI*-CSa2, found so far only in Togo, and three *COI*-RS haplotypes made up of *COI*-RSa2 found in 25 specimens spread over eight locations and single *COI*-RSa3 and *COI*-RSa4 specimens from Togo and Kenya, respectively (Table 2).

**Table 2:** COIB haplotype data. Data from earlier study^36^ identified by asterisk.

Collection	COI-CS types		COI-RS types				
	CSa1	CSa2	RSa1	RSa2	RSa3	RSa4	Total
GHA	10	0	25	1	0	0	36
TOGa*	52	1	23	2	0	0	78
TOGb	211	3	112	13	1	0	340
STP*	13	0	5	0	0	0	18
CHA	3	0	15	0	0	0	18
nDRC*	11	0	10	0	0	0	21
sDRC*	16	0	46	5	0	0	67
CAR	8	0	19	1	0	0	28
BUR*	16	0	22	0	0	0	38
KEN*	2	0	42	1	0	1	46
TAN*	5	0	59	1	0	0	65
ZAM	10	0	40	1	0	0	51
SAf	8	0	81	0	0	0	89
Mean frequency	0.33	<0.01	0.64	0.02	<0.01	<0.01	

**Figure 4:**
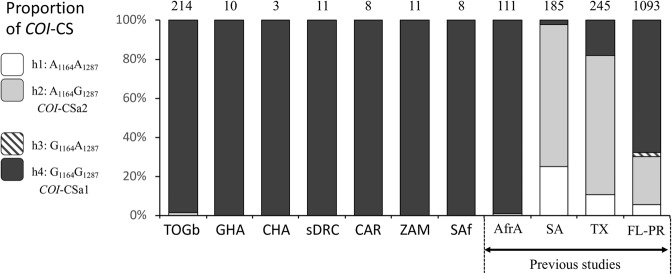
Frequency distributions of the COIB h-haplotypes, h1–4, for select locations in the Western Hemisphere and Africa. Numbers above columns indicate the number of *COI*-CS specimens. For Previous studies: AfrA = Africa^36^, SA = South America^30,43,52^, TX = Texas^32^, FL-PR = Florida and Puerto Rico^31,32^.

Polymorphisms at two sites in COIB, the strain-diagnostic mCOI1164D and mCOI1287R, subdivide the *COI*-CS group into four configurations (h1 = A_1164_A_1287_, h2 = A_1164_G_1287_, h3 = G_1164_A_1287_, h4 = G_1164_G_1287_) that differ regionally in their Western Hemisphere distributions^29,30^. The h4 configuration is the majority form in Florida and Puerto Rico and is represented in Africa by the *COI*-CSa1 haplotype that predominates at all African sites (Fig. 4, Table 2). The h2 configuration, which is found in over 75% of corn-strain specimens from Texas and South America, has so far only been found in the Togo collections (*COI*-CSa2) where it represents 1% (4/376) of the *COI*-CS specimens. The h1 and h3 configurations, which are in the minority in the Western Hemisphere, has yet to be reported in Africa.

### Characterization of *Tpi* haplotypes

The African populations consist of only three TpiE4 haplotypes, two TpiC variants (TpiCa1 and TpiCa2) and a single rice-strain haplotype, TpiRa1 (Fig. 1b, Table 3). The TpiRa1 haplotype is unusual in that it differs from the consensus Western Hemisphere TpiR sequence at four sites (Supplementary Fig. S3). Three sites, (gTpi129C, gTpi144G, and gTpi180C) are not frequently polymorphic in the Western Hemisphere, while one (gTpi165Y) exhibits strain-specific variation. The TpiRa1 haplotype has not yet been found in Western Hemisphere collections^35,36^. The corn-strain TpiCa1 and TpiCa2 differ at sites gTpi192Y and gTpi198Y, with both variants common in the Western Hemisphere. The three African TpiE4 haplotypes were observed to form three classes of heterozygotes noted as TpiC-YY = TpiCa1/TpiCa2, TpiH-CC = TpiCa1/TpiRa1, and TpiH-YY = TpiCa2/TpiRa1 (Fig. 1b, Table 3).

**Table 3:** TpiE4 haplotype data. Results from earlier study^36^ identified by asterisk.

Location	TpiE4 haplotypes (observed specimens)						TpiE4 haplotypes (estimated chromosomes)^a^		
	Ca1	Ca2	Ra1	C-YY	H-CC	H-YY	Ca1_adj_	Ca2_adj_	Ra1_adj_
TOGa*	16	17	2	24	8	9	56	59	20
TOGb	70	47	3	59	13	9	212	162	28
STP*	6	3	0	6	1	2	16	13	3
GHA	16	6	2	7	3	1	34	17	7
CHA	7	1	0	4	3	3	18	9	6
nDRC*	12	0	0	9	3	0	30	9	3
sDRC*	27	8	2	19	5	1	65	32	9
CAR	12	4	0	6	4	2	28	14	6
BUR*	21	2	0	10	4	0	46	13	4
KEN*	12	2	1	13	3	8	34	24	13
TAN*	35	2	0	13	5	3	71	19	8
ZAM	22	13	0	10	3	1	46	31	4
SAf	34	7	2	23	1	1	75	35	5
Mean frequency	0.43	0.13	0.01	0.28	0.09	0.07	0.59	0.31	0.10

Adjusted haplotype numbers based on extrapolated genotypes of heterozygotes: C-YY = Ca1/Ca2, H-CC = Ra1/Ca1, H-YY = Ra1/Ca2.

^a^Adjusted values, see Methods.

Although the TpiC marker as a whole showed no regional differences in distribution (Fig. 2b), the TpiCa1 haplotype occurred significantly more frequently in the collections from Togo and São Tomé and Príncipe compared to those from other African locations (Fig. 2c). This pattern is consistent with an earlier but more limited study^36^, and is similar to that observed for *COI*-CS frequency (Fig. 2a).

### *Tpi* intron comparisons

The differences in the Africa TpiRa1 sequence relative to those found in the Western Hemisphere brings into question whether it truly represents a rice-strain defining haplotype. To address this issue and to find additional TpiC variants we sequenced a 172-bp fragment (TpiI4) from the adjacent intron that was previously shown to be highly polymorphic in the Western Hemisphere populations^39^. A total of 854 specimens from 11 African nations were analyzed, including all 11 TpiRa1 samples. Approximately half (405) of the specimens tested were heterozygous for the TpiI4 segment as indicated by overlapping sequence chromatographs. Out of the 449 remaining unambiguous sequences six unique TpiI4 sequences were found (Table 4). TpiRa1 was associated with a single TpiI4 sequence (TpiI4Ra1). The TpiCa1 exon haplotype was linked to two intron sequences, TpiCa1a and TpiCa1b, with the latter differentiated primarily by a 200-bp insertion (Supplementary Fig. S3). The TpiCa2 exon haplotype was associated with three intron variants, TpiCa2a-c, that differed from TpiCa1a by between 5–11 single base changes.

**Table 4:** TpiI4 haplotype data.

	Number of TpiI4 haplotypes						
Collection	Ca1a	Ca1b	Ca2a	Ca2b	Ca2c	Ra1a	Heterozygotes
TOGa	16	0	11	2	0	2	46
TOGb	88	0	30	10	1	3	116
STP	6	0	2	0	0	0	10
GHA	17	0	1	0	0	1	16
CHA	7	0	1	0	0	0	10
nDRC	6	0	0	0	0	0	6
sDRC	26	0	4	3	0	2	25
CAR	13	0	4	0	0	0	11
BUR	18	0	1	0	0	0	16
KEN	10	0	1	1	0	1	27
TAN	27	0	1	1	0	0	26
ZAM	23	0	10	3	0	0	12
SAf	31	0	3	2	0	2	28
Mean frequency	0.41	0.00	0.07	0.02	<0.00	0.01	0.50

The TpiI4Ca1a haplotype was the most common found in Africa, making up 40% of all specimens, followed by TpiI4Ca2a (7%) and TpiI4Ca2b (2%), with most of the remainder found as heterozygotes (Table 4). The TpiI4Ca1b and TpiI4Ca2c sequences were each represented by a single specimen collected in Togo, while the 11 TpiI4Ra1 specimens were distributed in the collections from five African nations.

**Figure 5:**
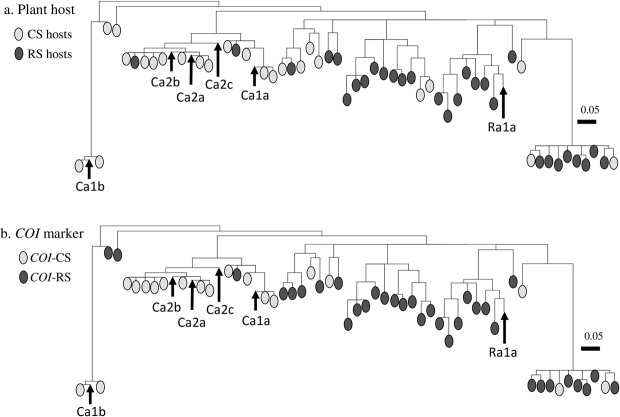
Phylogenetic tree inferred by using the Maximum Likelihood method and Tamura-Nei model^50^. The tree with the highest log likelihood (−1421.58) is shown. The six African TpiI4 intron variants were compared to 53 unique haplotypes found from 308 Western Hemisphere (Argentina, Brazil, Texas, and Florida) larvae collected from strain preferred host plants. (**a**) Phylogenetic tree color-coded for the host plant from which the specimen was collected. CS hosts = maize or sorghum, RS hosts = turf or pasture grasses. (**b**) Same phylogenetic tree coded for *COI*-based strain identification. *COI*-CS = corn-strain, *COI*-RS = rice-strain. Scale bar represents substitutions per site.

The African TpiI4 haplotypes were compared to a database of 53 unique TpiI4 sequences from the Western Hemisphere (Argentina, Brazil, Florida, Puerto Rico, and Texas) derived from a total of 308 larval specimens collected from either corn-strain (maize, sorghum, cotton) or rice-strain (pasture grasses, millet) host plants. A single phylogenetic tree was generated describing the genetic relationships between sequences and color coded to show the distribution of the African TpiI4 haplotypes relative to host plant and *COI* strain markers (Fig. 5).

The TpiRa1a haplotype clustered with a clade that was 100% comprised of sequences from larvae collected from rice-strain host plants (Fig. 5a) or expressing the rice-strain defining *COI*-RS marker (Fig. 5b). Similarly, the five TpiI4 haplotypes associated with the *Tpi*-defined corn-strain TpiCa1 and TpiCa2 exon sequences fell into clusters predominated by both corn-strain preferred host plants and the *COI*-CS marker. These findings strongly support the strain-identification based on the TpiE4 marker, specifically showing that the so far unique TpiRa1 exon variant is associated with an intron sequence that has a rice-strain identity based on plant host and the *COI* marker.

## Discussion

The most parsimonious mechanism for the invasion of fall armyworm into Africa is a single introduction followed by dispersion through natural and trade-related migration. The likelihood of establishment is most dependent on the size of the introductory population or propagule^40^, while also influenced by the characteristics of the species and the physical environment^41^. Studies on the invasive history of the fire ant, *Solenopsis invicta*, extrapolated a founding size of 9–20 unrelated queens for establishment in Mississippi, with the permanent population expressing as few as six mitochondrial haplotypes^42^. Fall armyworm entry into Africa on infested agricultural material in the form of egg masses or young larvae could mean a starting population of as many as a hundred or more individuals, which would be in the range projected for fire ant establishment in Mississippi. There are several observations that are consistent with this invasion scenario.

A single founder population would be expected to represent a bottleneck that reduces genetic variation and therefore the number of *COI* and *Tpi* haplotypes present. Surveys of fall armyworm from western Africa^35^, eastern Africa^36^, and now central and southern Africa demonstrated genetic variation throughout the continent is very limited. From almost a thousand specimens from 11 African nations only three COIB variants were identified. This is consistent with recent findings from Uganda and South Africa that also reported few *COI* haplotypes^36,37^. Most compelling is the evidence from the highly variable TpiI4 intron segment for which we found 53 unique variants from 308 specimens in the Western Hemisphere but only six distinct African intron sequences from 740 specimens.

If the geographically distant Africa populations all arose recently from the same source, then they should share some similarities in the type and frequency of haplotypes. The degree of similarity will be dependent on the frequency, magnitude, and pattern of the dispersal behavior. Consistent and extensive migration on a continental scale will tend toward genetic homogeneity while more limited mobility will create regional heterogeneity due to stochastic events such as genetic drift and population bottlenecks.

There is an overall similarity in the *COI* and *Tpi* composition of the different African populations consistent with their having a common origin. The same haplotypes predominated ( > 90%) in all locations, i.e., *COI*-CSa1 and *COI*-RSa1 (Table 2), the TpiE4 haplotypes TpiCa1 and TpiCa2 (Table 3), and the TpiI4 haplotypes TpiI4Ca1a and TpiI4Ca2a (Table 4). In addition, the single TpiR variant found in Africa, TpiRa1, has a broad distribution on the continent, having been found in Togo, Ghana, the Democratic Republic of the Congo, Kenya, and South Africa (Table 3). Yet it has so far not been detected in a survey of several hundred specimens from throughout the Western Hemisphere, suggesting that this is a relatively rare haplotype. These observations make it unlikely that the broad TpiRa1 geographical range is due to multiple independent introductions from different source populations. Finally, the discordant strain maker configuration *COI*-RS TpiC is a minority genotype in the Western Hemisphere but predominates in most of Africa (with the exception of Togo and São Tomé and Príncipe). The reason for this pattern is unknown but a single set of stochastic events producing the discordance followed by its dispersion to the rest of the continent seems a simpler explanation than multiple incursions with each coincidentally producing a majority with the same discordant configuration.

There is some evidence of genetic structure in the African populations, though the low genetic variability in the markers so far tested limit such analyses. We now have two years of data for Togo representing several hundred specimens from both larval collections and pheromone traps that together with one season of data from São Tomé and Príncipe show statistically significant differences in the frequency of *COI* and *Tpi* haplotypes from the rest of the continent (Figs 3 and 4). An earlier study with more limited data suggested the possibility that haplotype frequency differences followed an east-west axis, with the most western collections in Togo and São Tomé and Príncipe differing from the most eastern specimens from Kenya and Tanzania^36^. However, we found that fall armyworm from Ghana, which lies to the west of Togo, exhibits haplotype frequency patterns more similar to those found in central, eastern, and now southern Africa. Why Togo and São Tomé and Príncipe fall armyworm should differ from other parts of Africa is unknown as we know of no obvious differences in agricultural practices or habitat with the other surveyed locations. An explanation will require additional surveys of the region and should include methods to uncover more extensive genetic variation to make possible more sophisticated analysis of genetic structure and isolation over distance. The fact that such differences are observed even with the limited genetic markers at hand is an indication that fall armyworm migration in Africa may not be of sufficient frequency or magnitude to homogenize regional populations with respect to haplotype frequencies.

Two characteristics of fall armyworm strain behavior in the Western Hemisphere are that they are sympatric and are capable of productive interstrain mating^17,21,27^ despite the existence of hybridization barriers^8,23,25,26,28^. There are numerous observations that the two strains can be found in collections from a single plant host^19,21,22,43^, making it plausible that both were present in the propagule introducing fall armyworm to Africa. If so, that small initial mating population would be expected to exhibit a higher than normal level of interstrain hybridization, an example of admixture frequently observed during invasive events^44,45^. In addition, small breeding populations are often associated with inbreeding depression due to the higher likelihood of deleterious mutations becoming homozygous^46^. Under such conditions, interstrain hybrids could have a significant fitness advantage and become over-represented in the invasive population, leading ultimately to the loss of one or both strains in favor of more novel hybrid genotypes.

Events of this type could explain the extensive disagreement between the *COI* and *Tpi* strain markers observed in Africa fall armyworm populations. The strong bias for the rice-strain *COI*-RS type is inconsistent with the corn and sorghum host plants from which the Africa collections were made and contrasts with the predominance (>90%) of the corn-strain defining *Tpi*C marker in the same collections. The mitochondrial *COI* and nuclear *Tpi* genes are not physically linked so separation of the markers can occur from a single cross and thereafter segregate independently if strain identity is compromised. Population bottlenecks and random loss of genetic variation could then lead to the haplotype profiles currently observed. Testing this possibility will require more extensive genetic analysis as well as physiological and behavioral comparisons with the Western Hemisphere fall armyworm strains. If the African fall armyworms are primarily (or perhaps even entirely) interstrain hybrids their behaviors with respect to host plant preferences, mating behavior, and resistance may differ significantly from that characterized for Western Hemisphere populations that retain strain integrity. This could have important ramifications to the design and effectiveness of mitigation efforts.

In summary, genetic “snapshot” of fall armyworm populations spanning sub-Saharan Africa is presented that describes the situation two years after the first detection of the pest in 2016. This will be an important resource for future studies on how fall armyworm distributions may change as the invasive populations continue to equilibrate to their new environment. We believe the observed combination of low numbers of haplotypes, regional similarities in haplotype composition, regional differences in haplotype frequencies, and evidence of excessive interstrain hybridization is most parsimoniously explained by a single introduction followed by rapid dispersion through natural and trade-related processes, which while geographically extensive is so far not of a magnitude able to homogenize widely separated populations.

## Methods

### Specimen collections and DNA preparation

Specimens were obtained as adult males from pheromone traps in Togo maize (corn) fields or larvae from maize or sorghum plants at various locations in Chad, the Central African Republic, South Africa, Zambia, and Ghana in 2017 (Table 1). Additional specimens from previously described Democratic Republic of the Congo collections were analyzed^36^. These were from the Haut-Katanga province and were pooled with the earlier data from the same location (sDRC), which were separately analyzed from more northern collections (nDRC). Collected specimens were stored either air-dried or in ethanol at room temperature. A portion of each specimen was excised and homogenized in a 5-ml Dounce homogenizer (Thermo Fisher Scientific, Waltham, MA, USA) in 800 µl Genomic Lysis buffer (Zymo Research, Orange, CA, USA) and incubated at 55 °C for 5–30 min. Debris was removed by centrifugation at 10,000 rpm for 5 min. The supernatant was transferred to a Zymo-Spin III column (Zymo Research, Orange, CA, USA) and processed according to manufacturer’s instructions. The DNA preparation was increased to a final volume of 100 µl with distilled water. Genomic DNA preparations of fall armyworm samples from previous studies were stored at −20 °C. Species identity was initially determined by morphology and confirmed by sequence analysis of the COIB region. All specimens used had COIB sequences that differed by no more than a single site to haplotypes found in Western Hemisphere fall armyworm (Supplementary Fig. S2).

### PCR amplification and DNA sequencing

PCR amplification for all segments was performed in a 30-µl reaction mix containing 3 µl 10X manufacturer’s reaction buffer, 1 µl 10 mM dNTP, 0.5 µl 20-µM primer mix, 1 µl DNA template (between 0.05–0.5 µg), 0.5 unit Taq DNA polymerase (New England Biolabs, Beverly, MA). The thermocycling program was 94 °C (1 min), followed by 33 cycles of 92 °C (30 s), 56 °C (45 s), 72 °C (45 s), and a final segment of 72 °C for 3 min. Typically 96 PCR amplifications were performed at the same time using either 0.2-ml tube strips or 96 well microtiter plates. All primers were obtained from Integrated DNA Technologies (Coralville, IA). Amplification of the COIB segment used the primer pair *891F* (5′-TACACGAGCATATTTTACATC-3′) and *1472R* (5′-GCTGGTGGTAAATTTTGATATC-3′) to produce a 603-bp fragment. Amplification of the *Tpi* exon segment used the primers *412F* (5′-CCGGACTGAAGGTTATCGCTTG-3′) and *850R* (5′-AATTTTATTACCTGCTGTGG-3′) to produce a fragment containing most of the fourth exon with an approximate length of 199 bp. The adjacent TpiI4 intron segment was amplified using primers *412F* and *1140R* (5′-GCGGAAGCATTCGCTGACAACC-3′) to produce a variable length fragment due to insertion and deletion mutations.

For fragment isolations, 6 µl of 6X gel loading buffer was added to each amplification reaction and the entire sample run on a 1.8% agarose horizontal gel containing GelRed (Biotium, Hayward, CA) in 0.5X Tris-borate buffer (TBE, 45 mM Tris base, 45 mM boric acid, 1 mM EDTA pH 8.0). Fragments were visualized on a long-wave UV light box and manually cut out from the gel. Fragment isolation was performed using Zymo-Spin I columns (Zymo Research, Orange, CA) according to manufacturer’s instructions. The University of Florida Interdisciplinary Center for Biotechnology (Gainesville, FL) and Genewiz (South Plainfield, NJ) performed the DNA sequencing.

DNA alignments and consensus building were performed using MUSCLE (multiple sequence comparison by log-expectation), a public domain multiple alignment software incorporated into the Geneious Pro 10.1.2 program (Biomatters, New Zealand, http://www.geneious.com)^47^. Phylogenetic trees were graphically displayed in a neighbor-joining (NJ) tree analysis also included in the Geneious Pro 10.1.2 program^48^. Maximum Likelihood method based on the Tamura-Nei model was done using MEGA^49,50^.

### Characterization of the *CO1* and *Tpi* gene segments

The genetic markers are all single nucleotide substitutions. Sites in the *COI* gene are designated by an “m” (mitochondria) while *Tpi* sites are designated “g” (genomic). This is followed by the DNA name, number of base pairs from the predicted translational start site (*COI*), 5′ start of exon (*Tpi*), or 5′ start of the intron (TpiI4) and the nucleotides observed using IUPAC convention (R: A or G, Y: C or T, W: A or T, K: G or T, S: C or G, D: A or G or T).

The *COI* markers are from the maternally inherited mitochondrial genome. The COIB segment was amplified by *CO1* primers 891 F and 1472 R and used to determine host strain identity and determine the region-specific haplotypes. Sites mCOI1164D and mCOI1287R in Western Hemisphere populations identify a single rice-strain, T_1164_A_1287_, and four corn-strain configurations, A_1164_A_1287_ (h1), A_1164_G_1287_ (h2), G_1164_A_1287_ (h3), G_1164_G_1287_ (h4).

Variants in the TpiE4 exon segment can also be used to identify host strain identity with results generally comparable with the *CO1* marker^17^. The gTpi183Y site is on the fourth exon of the predicted *Tpi* coding region and was PCR amplified using the *Tpi* primers 412F and 1140R. The C-strain allele (*Tpi*C) is indicated by a C_183_ and the R-strain (*Tpi*R) by T_183_^17^. The *Tpi* gene is located on the *Z* sex chromosome that is present in one copy in females and two copies in males. Since males can be heterozygous for *Tpi*, there is the potential for the simultaneous display of both alternative nucleotides at *Tpi*_183_ (denoted as *Tpi*H), which would be indicated by an overlapping C and T DNA sequence chromatograph^27^.

The TpiI4 intron segment was sequenced used primers 412F for the initial sequencing reaction and 1140R for 2^nd^ strand sequence confirmation when needed in cases of ambiguity. Intron length is variable because of insertions and deletions. Based on the TpiC consensus sequence (identical to TpiCa1a) the TpiI4 segment is a 162-bp fragment from intron nucleotide 10 to 172 (Supplementary Fig. S2). This segment was chosen for analysis because it empirically had the most consistent intron sequence quality with the given primers, thereby facilitating analysis of a large number of specimens. One sites, gTpiI4[131]R, consistently gave poor signal and high background. Given this ambiguity, the consensus nucleotide (G_128_) was assumed.

Sequence data were available for the TpiI4 segment for fall armyworm larvae collected from either corn-strain or rice-strain preferred host plants from four nations or states. Sequences for each location were filtered for duplicates and hybrids. The remaining sequences were used for the phylogenetic tree analysis. These represent distinct intron haplotypes. The locations include (# total sequences, #distinct haplotypes) Argentina (116, 14), Brazil (96, 17), Florida (72, 19), and Texas (24, 3).

### Calculation of haplotype numbers

The mitochondrial *COI* markers are calculated as the number of specimens exhibiting the *COI* haplotypes (Table 2). Because *Tpi* is a sex-linked nuclear gene, heterozygotes are possible in male fall armyworm. The genotypes of heterozygotes of TpiE4 segment alleles can be unambiguously determined, with TpiC-YY = TpiCa1/TpiCa2, TpiH-CC = TpiRa1/TpiCa1, and TpiH-YY = TpiRa1/TpiCa2. When individual haplotypes could be extrapolated from heterozygotes, the data was adjusted. The Togo pheromone trap collections (TOGb) are all male so all have two *Tpi* genes. The adjusted number of haplotypes was calculate using the following equations: Number of TpiCa1 = 2 × (TpiCa1 specimens) + TpiC-YY specimens + TpiH-CC specimens; TpiCa2 = 2 × (TpiCa2) + TpiC-YY + TpiH-YY; TpiRa1 = 2 × (TpiRa1) + TpiH-CC + TpiH-YY (Table 3). In the case of the larval collections the genders of the individual specimens were unknown, so unambiguous haplotypes could be homozygous (2 *Tpi* copies) or hemizygous (one gene). A 1:1 sex ratio was assumed so that the average number of *Tpi* genes per specimen is given as 1.5, *i*.*e*., (2 in males + 1 in females)/2. Calculations of larval haplotype numbers used the same equations as with pheromone traps except that the number of specimens with an unambiguous haplotype was multiplied by 1.5 instead of 2.

In the case of the TpiI4 haplotypes, several of the heterozygotes could be due to multiple haplotype combinations. Because of this ambiguity only the number of specimens exhibiting an unambiguous haplotype was reported, along with the total number of heterozygous TpiI4 specimens (Table 4).

### Accession codes

MH726218 - MH726361.

## Supplementary information

Supplementary information

## Data Availability

All data generated or analyzed during this study are included in this published article. The sequences used for the phylogenetic analysis are deposited into GenBank and included in (MH726218 - MH726361).
