# Supplementary material for: Genetic comparisons of fall armyworm populations from 11 countries spanning sub-Saharan Africa provide insights into strain composition and migratory behaviors
Source: Sci Rep. 2019 Jun 5;9:8311. doi: 10.1038/s41598-019-44744-9 (PMC6549444; doi:10.1038/s41598-019-44744-9)
Supplement: Supplementary file 1 — Supplementary information [file 41598_2019_44744_MOESM1_ESM.pdf]

Supplementary Information

Genetic comparisons of fall armyworm populations from 11 countries spanning sub-Saharan Africa provide insights into strain composition and migratory behaviors.

Rodney N. Nagoshi[1*], Georg Goergen[2], Hannalene Du Plessis[3], Johnnie van den Berg[3], and Robert L. Meagher[1]

[1]Center for Medical, Agricultural and Veterinary Entomology, United States Department of Agriculture-Agricultural Research Service, Gainesville, Florida, United States of America.

[2]International Institute of Tropical Agriculture (IITA), Cotonou, Benin

[3]Unit for Environmental Sciences and Management, North-West University, Potchefstroom, South Africa

* Corresponding author
E-mail: rodney.nagoshi@ars.usda.gov

Supplementary Figure legends

Supplementary Figure S1. Map of collection sites in Africa described in Table 1.

Supplementary Figure S2. Description of the COIB polymorphisms and the haplotypes observed in African fall armyworm. The consensus Western Hemisphere *COI*-CS (WHCOI-CS) and *COI*-RS (WHCOI-RS) sequences are in dark bold. The six COIB haplotypes observed in Africa are listed with two in the *COI*-CS and four in the *COI*-RS categories. Strain-specific polymorphisms are indicated by bold lettering. Differences within strain categories are denoted by dark bold. Sites polymorphic in the consensus are indicated by the IUPAC symbol R, which indicates a G or A alternative. Asterisk identifies site used for strain identity.

Supplementary Figure S3. Descriptions of the *Tpi* gene segments used in the fall armyworm analysis. Numbered locations indicate relevant polymorphic sites. A, haplotype sequences in the TpiE4 segment are shown with WHTpiR and WHTpiC the consensus sequences for the strain-specific *Tpi* haplotypes derived from Western Hemisphere data. Asterisks identify strain-specific polymorphic sites defined by Western Hemisphere populations. Note that site 165 is strain-specific in the Western Hemisphere but not in Africa. Dark and bold lettering indicate differences from the reference sequences. B, the six TpiI4 haplotypes found in Africa. Letters in dark bold identify polymorphisms. Site 131 was associated with consistently poor sequence quality and so was assumed to be the consensus G for all specimens. Dark triangle indicates insertion site in TpI4Ca1b of a 200-bp insertion.

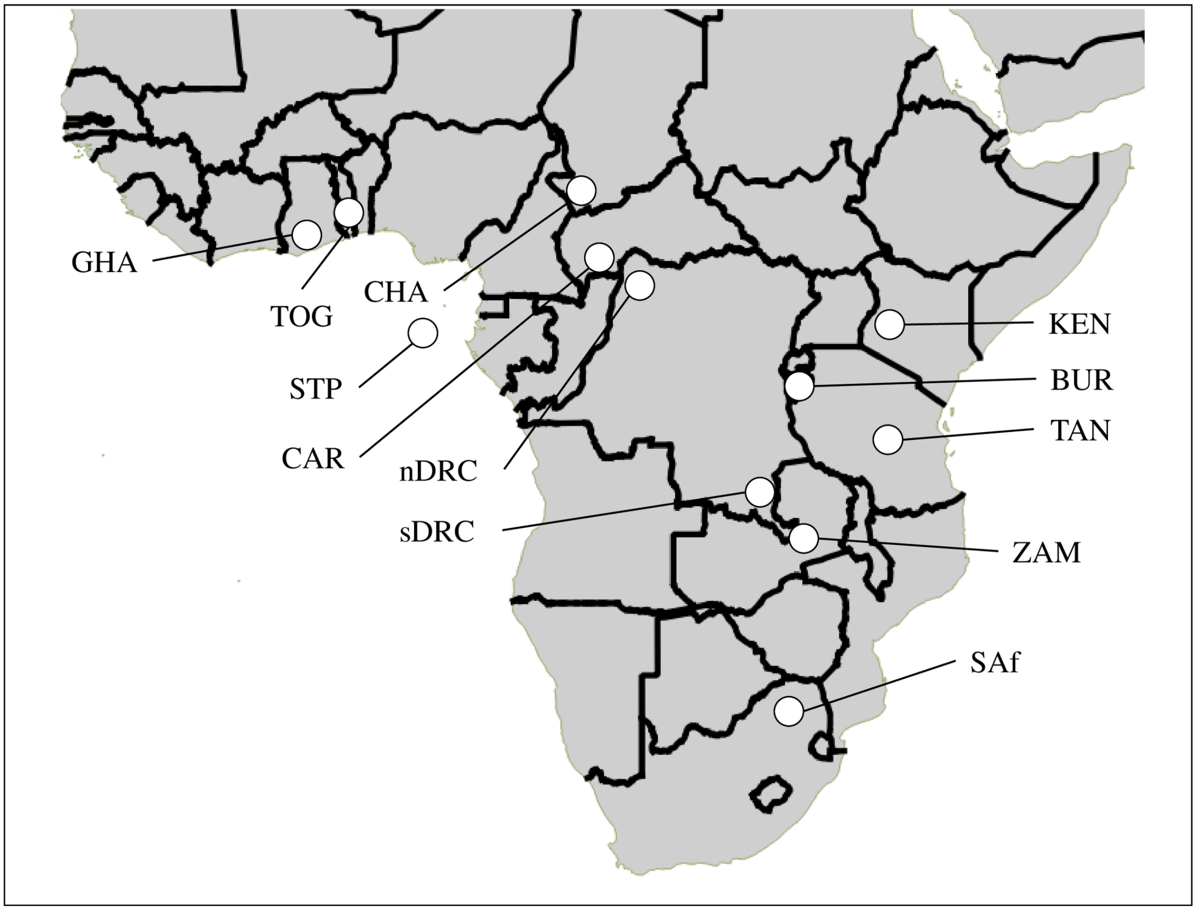

Supplementary Figure S1

```
                1125                                       1164*       1176
                |                                          |           |
WHCOI-CS  CATTTCCATTATGTTTTATCAATAGGAGCTGTATTTGCTATTTTAGGRGGATTTATTCAT
COI-CSa1  CATTTCCTTTATGTTTTATCAATAGGAGCTGTATTTGCTATTTTAGGGGGATTTATTCAT
COI-CSa2  CATTTCCTTTATGTTTTATCAATAGGAGCTGTATTTGCTATTTTAGGAGGATTTATTCAT
WHCOI-RS  CATTTCCACTATGTTTTATCAATAGGAGCTGTATTTGCTATTTTAGGTGGATTTATTCAC
COI-RSa1  CATTTCCACTATGTTTTATCAATAGGAGCTGTATTTGCTATTTTAGGTGGATTTATTCAC
COI-RSa2  CATTTCCATTATGTTTTATCAATAGGAGCTGTATTTGCTATTTTAGGTGGATTTATTCAC
COI-RSa3  CATTTTCACTATGTTTTATCAATAGGAGCTGTATTTGCTATTTTAGGTGGATTTATTCAC
COI-RSa4  CATTTCCACTATGTTTTATCAATAGGAGCTGTATTTGCTATTTTAGGTGGATTTATTCAC

               1182           1197                1216
               |              |                   |
WHCOI-CS  TGATACCCATTATTTACTGGGTTATCTTTAAATCCTTATTTATTAAAAATTCAATTTTTT
COI-CSa1  TGATACCCATTATTTACTGGGTTATCTTTAAATCCTTATTTATTAAAAATTCAATTTTTT
COI-CSa2  TGATACCCATTATTTACTGGGTTATCTTTAAATCCTTATTTATTAAAAATTCAATTTTTT
WHCOI-RS  TGATATCCATTATTTACTGGATTATCTTTAAATCCTTAATTATTAAAAATTCAATTTTTT
COI-RSa1  TGATATCCATTATTTACTGGATTATCTTTAAATCCTTAATTATTAAAAATTCAATTTTTT
COI-RSa2  TGATATCCATTATTTACTGGATTATCTTTAAATCCTTAATTATTAAAAATTCAATTTTTT
COI-RSa3  TGATATCCATTATTTACTGGATTATCTTTAAATCCTTAATTATTAAAAATTCAATTTTTT
COI-RSa4  TGATATCCATTATTTACTGGATTATCTTTAAATCCTTCATTATTAAAAATTCAATTTTTT

                                                       1287
                                                       |
WHCOI-CS  ATTATATTTATCGGAGTAAATTTAACTTTCTTCCCACAACATTTTTTAGGRTT
COI-CSa1  ATTATATTTATCGGAGTAAATTTAACTTTCTTCCCACAACATTTTTTAGGGTT
COI-CSa2  ATTATATTTATCGGAGTAAATTTAACTTTCTTCCCACAACATTTTTTAGGGTT
WHCOI-RS  ATTATATTTATCGGAGTAAATTTAACTTTCTTCCCACAACATTTTTTAGGATT
COI-RSa1  ATTATATTTATCGGAGTAAATTTAACTTTCTTCCCACAACATTTTTTAGGATT
COI-RSa2  ATTATATTTATCGGAGTAAATTTAACTTTCTTCCCACAACATTTTTTAGGATT
COI-RSa3  ATTATATTTATCGGAGTAAATTTAACTTTCTTCCCACAACATTTTTTAGGATT
COI-RSa4  ATTATATTTATCGGAGTAAATTTAACTTTCTTCCCACAACATTTTTTAGGATT
```

Supplementary Figure S2

```
A. TpiE4
                 129               144                  165* 168*        180 183*      192   198
                 |                 |                       \   /           \  /          |     |
WHTpiR:  TGGCAGCAACTGGGACAAGGTCGTACTAGCTTATGAACCTGTCTGGGCTATTGGCACTGGAAAGACYGCCACYCCA
TpiRa1:  TGGTAGCAACTGGGACAAAGTCGTACTAGCTTATGAACCCGTCTGGGCTATTGGGACTGGAAAGACCGCCACCCCA

WHTpiC:  TGGCAGCAACTGGGACAAGGTCGTACTAGCTTATGAACCCGTTTGGGCTATTGGCACCGGAAAGACYGCCACYCCA
TpiCa1:  TGGCAGCAACTGGGACAAGGTCGTACTAGCTTATGAACCCGTTTGGGCTATTGGCACCGGAAAGACCGCCACCCCA
TpiCa2:  TGGCAGCAACTGGGACAAGGTCGTACTAGCTTATGAACCCGTTTGGGCTATTGGCACCGGAAAGACTGCCACTCCA
```

```
B. TpiI4
                                28 29 31     38               53 55  58            70      77  80   84  87
                                 \  /  /     |                 \  /  |             |       |   |    |   |
TpiRa1a  TTAATTTGTTTTATAAAAATGCAGGATAAAAAATG---CATGCAAATCTTTATTGGTATTAAAAGTTTACTTCCATAATCTTG
TpiCa1a  TTAATTTGTTTTAT--AAATAAACGATAAACAATGATACATGCAAATCTTGATTGGTATTAAAAGTTTACTTACATGATCTTG
TpiCa1b  TTAATTTGTTTTAT--AAATAAACGATAAACAATGATACATGCAAATCTTGATTGGTATTAAAAGTTTACTTACATGATCTTG
TpiCa2a  TTAATTTGTTTTAT--AAATAAAGGATAAAAAATGATACATGCAATTATTTATTGGTATTAACAGTTTATTTACATGATTTTG
TpiCa2b  TTAATTTGTTTTAT--AAATAAAGGATAAAAAATGATACATGCAATTATTTATTGGTATTAACAGTTTATTTACATGATTTTG
TpiCa2C  TTAATTTGTTTTAT--AAATAAAGGATAAAAAATGATACATGCAATTATTTATTGGTATTAACAGTTTATTTACATGATCTTG

              96                                      131 136                148     155         167 171
              |                                         |   |                  |       |           |   |
TpiRa1a  AACTGTGCTTATCTAGTAAATAGTGTTTAGAATTTAATCAGAATTGGTTTCAGTAATCAGTATGCTATTTAAAACCCATATG
TpiCa1a  AACTGTGCTTATCTAGTAAATAGTGTTTAGAATTTAATCAGAATTGGTTTCAGTAATCAGTATGTTATTTAAAACCTATAGG
TpiCa1b  AACTGTGCTTATC▼TAGTAAATAGTGTTTAGAATTTAATCAGAATTAGTTTCAGTAATCAGTATGTTATTTAAAACCTATAGG
TpiCa2a  AACTGCGCTTATCTAGTAAATAGTGTTTAGAATTTAATCAGAATTGGTTTCAGTAATAAGTATGTTATTTAAAACCTATAGG
TpiCa2b  AACTGCGCTTATCTAGTAAATAGTGTTTAGAATTTAATCAGAATTGGTTTCAGTAATCAGTATGTTATTTAAAACCTATAGG
TpiCa2C  AACTGTGCTTATCTAGTAAATAGTGTTTAGAATTTAATCAGAATTGGTTTCAGTAATCAGTATGTTATTTAAAACCTATAGG

TpI4Ca1b insertion(▼):  TCTATTATATAAAAATAAGTCGGGTTTTCCTTCCTGACGCTATAACTCCAGAATGCACGAACCGATT
                        TCCACGGTTTTGCATCCGTTGGAAAGGTCTCGGGCTCCGTGAGGTTTATAGCAAAGAAAATTCCGGA
                        TTAAAAAAAAAGCACGAGGACACTGAAAATCATTGGTGGCGAAACGGGGTTCGCCGGGTTTGCTAG
```

Supplementary Figure S3
